# Sorption-Enhanced Dry Reforming of Methane in a DBD Plasma Reactor for Single-Stage Carbon Capture and Utilization

**DOI:** 10.1021/acssuschemeng.4c02502

**Published:** 2024-07-06

**Authors:** Rani Vertongen, Giulia De Felice, Huub van den Bogaard, Fausto Gallucci, Annemie Bogaerts, Sirui Li

**Affiliations:** † Research Group PLASMANT, Department of Chemistry, University of Antwerp, Universiteitsplein 1, Antwerp 2610, Belgium; ‡ Research Group Inorganic Membranes and Membrane Reactors, Sustainable Process Engineering, Department of Chemical Engineering and Chemistry, Eindhoven University of Technology, De Rondom 70, Eindhoven 5612 AP, The Netherlands

**Keywords:** plasma, dry reforming of methane, dielectric barrier discharge, sorbent, carbon capture and utilization, zeolite

## Abstract

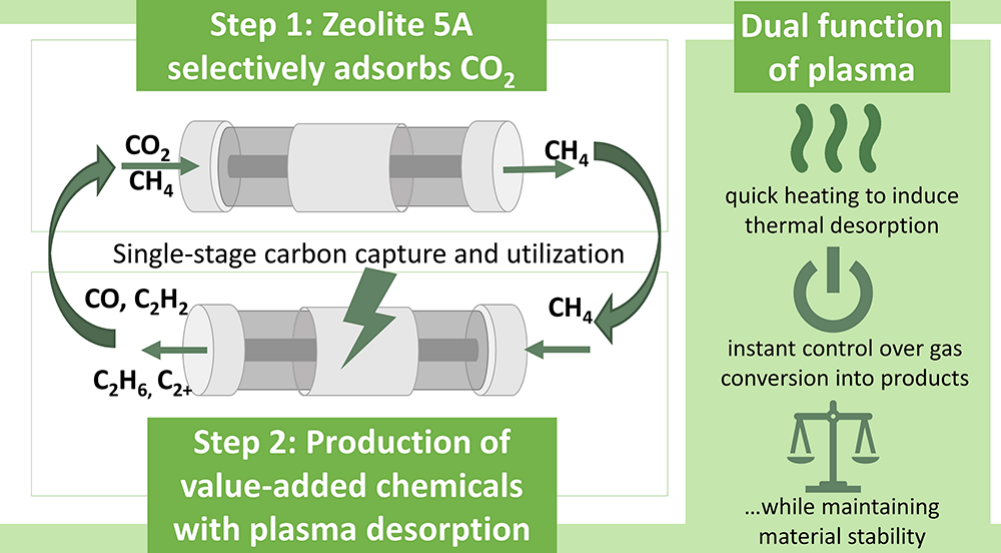

Plasma–sorbent systems are a novel technology for single-stage carbon capture and utilization (CCU), where the plasma enables the desorption of CO_2_ from a sorbent and the simultaneous conversion to CO. In this study, we test the flexibility of a plasma–sorbent system in a single unit, specifically for sorption-enhanced dry reforming of methane (DRM). The experimental results indicate the selective adsorption of CO_2_ by the sorbent zeolite 5A in the first step, and CH_4_ addition during the plasma-based desorption of CO_2_ enables DRM to various value-added products in the second step, such as H_2_, CO, hydrocarbons, and the byproduct H_2_O. Furthermore, our work also demonstrates that zeolite has the potential to increase the conversion of CO_2_ and CH_4_, attributed to its capability to capture H_2_O. Aside from the notable carbon deposition, material analysis shows that the zeolite remains relatively stable under plasma exposure.

## Abstract 2

The plasma−sorbent combination is a flexible system to convert greenhouse gases into value-added chemicals.

## Highlights

- Value-added products formed by adsorption of CO_2_ and desorption in a CH_4_ plasma.
- Plasma heating contributes to the thermal desorption of CO_2_.
- Zeolite 5A is beneficial to DRM because it captures the byproduct H_2_O.
- No significant plasma-induced changes in surface area and morphology of sorbents.

## 1 Introduction

Global warming is a complex problem, and there is significant pressure for urgent action and meaningful change.^1^ In the transition to a more sustainable society, defossilization is crucial to reducing CO_2_ emissions in the chemical industry through electrification and carbon capture, utilization, and storage.^2,3^ Although carbon capture and storage (CCS) is most promising to decrease CO_2_ emissions effectively in the short term,^4,5^ large-scale projects are only just starting.^6^ Known limitations are the high cost of separation, enrichment, transportation of CO_2_, and the negative impact on ecology associated with physical storage.^7,8^ Alternatively, carbon capture and utilization (CCU) aims to apply the captured CO_2_ into products or convert it into value-added chemicals and fuels through electrified technologies.^9^ Not only does this decrease our dependence on fossil fuels, but also it can help to store the excess and uncertain supply of renewable electricity as stable chemical energy.^10^ However, many CCU processes still require multiple stages from the adsorption to the utilization, and possible steps in between stages, such as the transport and storage of CO_2_.^11^

One way to circumvent these steps is to apply single-stage CCU and reduce the overall process cost.^12^ More specifically, “single-stage” CCU refers to the integrated capture and utilization of CO_2_ in one process, particularly within a single reactor unit. For example, with adsorption-based carbon capture, CO_2_ is first captured from a dilute source such as flue gas (∼15 vol % CO_2_) or direct air capture (DAC).^13^ Then, the adsorbed CO_2_ is converted *in situ* while simultaneously regenerating the sorbent. The concept falls within the domain of “integrated carbon capture and utilization (ICCU)″, which has been described by Liu et al.,^14^ despite the potential ambiguity associated with the term “integrated” within the context of process design.

Various technologies have already been proposed when considering solely the conversion of CO_2_. The thermocatalytic approach typically applies a reducing atmosphere such as H_2_ or CH_4_ to enable generally high-temperature conversion with various catalysts.^12^ Alternative technologies are also gaining increasing attention, such as electro-,^15^ photo-,^16^ and plasma-catalytic^17^ conversion steps, thanks to their operation at ambient pressure and temperature. Plasma technology is a particularly flexible solution for CO_2_ conversion since it can be easily switched on/off with immediate production, and it does not rely on rare metal catalysts for good performance.^18^ The energetic electrons and reactive species in the plasma can activate stable molecules at ambient conditions, resulting in a wide range of gas conversion applications, as summarized by Bogaerts et al.^19^

However, in realistic applications, scenarios often involve the presence of contaminants or, conversely, low CO_2_ concentrations, necessitating enrichment through processes involving adsorbents, as seen in DAC. Several reported studies have focused on addressing these challenges, including not only the effect of impurities^20^ but also the combination with sorbents in plasma-based CCU in a one-stage^17,21−25^ and double-stage configuration.^26−30^ Yoshida et al.^21^ studied the desorption of CO_2_ when the sorbent material is placed inside the plasma zone. They even demonstrated faster desorption with plasma than with a thermal approach, and they attributed this effect to the interaction of the electrons and reactive species in the plasma with the sorbent material. Li et al.^22^ proposed CO_2_ capture with a hydrotalcite sorbent and plasma-based desorption and conversion in a dielectric barrier discharge (DBD) plasma reactor. By further optimizing the experimental conditions, they achieved a maximum single-pass conversion of 60%.^17^ They also tested the periodic operation of multiple reactors in parallel and in series for continuous operation, which resulted in the full conversion of CO_2_ into CO and O_2_ in the outlet stream.

Furthermore, the potential of plasma-based single-stage CCU can be extended beyond the splitting of CO_2_ for CO production, also to the production of value-added chemicals through CO_2_ hydrogenation or dry reforming of methane (DRM). Kaikkonen^23^ tested dual-functional materials for the reaction of adsorbed CO_2_ with H_2_ and found that the organic Hydrocell-resin had the highest adsorption capacity and a CO_2_ conversion of 15%. Despite this, there have been limited studies reported in this field thus far, particularly regarding DRM, which could be more complex due to its involvement in the capture and conversion of both CO_2_ and CH_4_. Gorky et al.^24^ investigated plasma-based desorption of CH_4_ and CO_2_ using MOF-177 as the sorbent. Notably, the gases were not mixed during the process in their study. In a subsequent study, various silicoaluminophosphate (SAPO) zeolites were employed, revealing that higher acidity or larger pore size contributes to a slower desorption rate.^25^ Nevertheless, for DRM in a plasma–sorbent system, many important aspects require more investigation, including the impact of plasma on the sorbent, desorption mechanisms, and potential reactions between CO_2_ and CH_4_.

To the best of our knowledge, no previous research has investigated DRM with a plasma–sorbent system for single-stage CCU. Despite the significant potential of this topic, the novel concept requires validation and a deeper understanding of the interaction between plasma and sorbents. Such studies would also benefit practical applications, particularly in improving the coupling of gas mixtures such as biogas^31^ and landfill gas^32^ to the plasma through the utilization of sorbents. Hence, this study focuses on experimentally investigating sorption-enhanced DRM using a plasma–sorbent system. Solid sorbents are combined with nonthermal plasma within a single DBD reactor. The research delves into plasma-induced desorption and conversion of CO_2_ and CH_4_ to demonstrate the single-stage CCU for DRM, with a discussion on possible underlying mechanisms.

## 2 Methods

### 2.1 Experimental Setup

**Figure 1:**
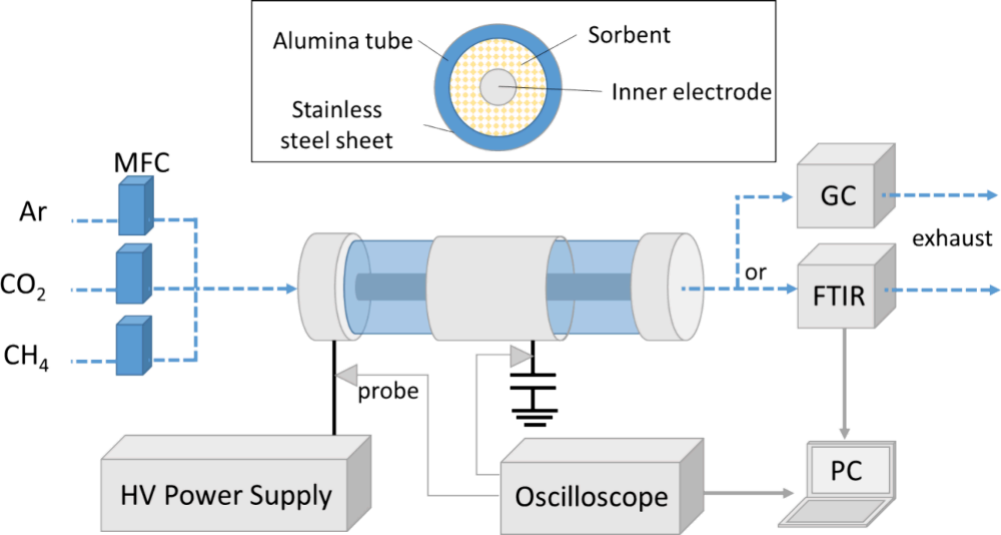
Experimental setup for plasma-induced CO_2_ desorption and conversion.

The experimental setup including the DBD reactor used in this study is shown in Figure 1; more details can be found in our previous publication.^17^ The dielectric barrier of the reactor was an alumina tube with external and internal diameters of 12.9 mm and 8.6 mm, respectively. A thin stainless-steel sheet (100 mm long) was placed around the tube and connected to the ground via a 100 nF capacitor. The inner electrode was a stainless-steel rod with a diameter of 6.7 mm, resulting in a discharge gap of 0.95 mm. The electrode was connected to the AC high-voltage power supply (AFS G155–150 K) fixed at 45 kHz. A four-channel oscilloscope (PicoScope 3405D, 100 MHz, 8-bit sampling rate of 1 G/s) was used to record the voltage waveforms. A 1:1000 high-voltage probe (Tektronix P6015A) was used to monitor the voltage (*V*) across the reactor. To measure the charge (*Q*) transferred during the plasma discharge, a 1:10 probe (Pico TA 131) measured the voltage across a 100 nF capacitor. To calculate the discharge power, both waveforms were recorded to form Lissajous figures.^33^

The inlet flow rate was controlled by mass flow controllers (Bronkhorst, calibrated at 0 °C and 1.013 bar) for Ar, CO_2_, and CH_4_. After the reactor, the outlet gas flowed to a Fourier transform infrared spectroscopy (FTIR) spectrometer (Agilent Technology, Cary 630) to analyze the gas composition as a function of time. The spectrometer has a gas cell with CaF_2_ windows, connected to a RED-SHIFT gas sampling system, and a scan resolution of 4 cm^–1^. All measurements were performed at room temperature (no external temperature control supplied to the reactor) and atmospheric pressure. The FTIR spectra were collected, and the concentrations of the components were calculated with the software Kinetic Pro and Microlab. An additional measurement was performed by connecting the outlet gas to an in-line gas chromatograph (GC, Thermo Scientific Trace 1300) equipped with two thermal conductivity detectors and a flame ionization detector. A small N_2_ flow (5 mL_*n*_/min) was added after the plasma reactor as an internal standard. This allowed the identification of C_1_–C_3_ hydrocarbons that overlap with the CH_4_ peaks in the FTIR spectrum and IR-inactive molecules such as H_2_. It is important to note that GC measurements require a longer duration for sampling and analysis (approximately 15 min per measurement in our case). This prohibits continuous measurements during the desorption stage, with short intervals between samples to capture the transient behavior of gas composition, as achievable with FTIR (10 s per measurement). Hence, we conducted GC measurements through a series of repeated experiments, where each measurement is taken at specific times after plasma ignition. To study the influence of water, a simple humidity meter (Extech Instruments Humidity Alert II 445815) was installed in the exhaust line. The relative humidity is measured, i.e., the actual amount of water vapor in the air compared to the total amount of vapor that can exist in the air at the current temperature.^34^ To study the outside reactor temperature, a small thermal camera (FLIR ONE Pro) was installed on the USB-c port of an Android smartphone. It was installed on a stand such that the camera could capture the warm region of the reactor.

We used 2.3 g of zeolite 5A beads (LTA type zeolite, mesh 8–12, Sigma-Aldrich) in this study, modified to a size of 250–355 μm. Pretreatment in plasma was performed to remove any ambient H_2_O and CO_2_ that might be adsorbed on the material (see Section 2.2 for an exact description of the conditions). This commercial molecular sieve is also commonly applied in industry and investigated for adsorption.^35,36^ In principle, it is possible to use other sorbents that have been reported in the literature.^37−39^ For example, Li and Gallucci^17^ previously used hydrotalcite pellets. Despite their good performance, this material requires more extensive pretreatment (up to 6000 s of plasma exposure) to remove the H_2_O that is inherently present in the structure. Furthermore, these pellets are relatively brittle compared to other commercial sorbents. We conducted pretests on zeolite 4A, the results of which are presented in the Supporting Information (SI), Section S1, revealing a low CO_2_ capacity. Therefore, we opted for zeolite 5A owing to its good stability and higher CO_2_ capacity. This test highlights the importance of sorbent choice, compared to the field of plasma catalysis, where adsorption and desorption have not been investigated thoroughly in typical studies. In addition, quartz particles within the same size range were packed and tested under the same experimental conditions. This control measurement served as a reference to quantify any delays in the measurement due to the volume of the lines and the stabilization time of the mass flow controllers.

To study the effect of plasma exposure, the material was characterized before and after plasma treatment. The morphology of the surface was investigated through scanning electron microscopy (SEM) with a Phenom Microscope ProX equipped with a backscattered electron detector (BSD) and a secondary electron detector (SED). The images were collected at several magnifications. Furthermore, the surface area and the pore volume were investigated through nitrogen physisorption at −196 °C with a TriStar 3000 Micromeritics, applying the Brunauer–Emmett–Teller (BET) plot method and the Barret–Joyner–Halenda (BJH) method.

### 2.2 Adsorption–Desorption Procedure

The general procedure is summarized in Table 1. All experiments were performed at atmospheric pressure, and no external heating or cooling was applied to the reactor, besides the influence of the plasma. Three repeated experiments were performed for each sorbent with the same sorbent sample; the small effect of the separate runs on the adsorption is displayed in the SI, Section S2.

**Table 1:** Overview of the General Adsorption–Desorption Procedure in the DBD Plasma Reactor at Atmospheric Pressure and Room Temperature (No External Temperature Control)

	time (s)	Ar (mL_*n*_/min)	CO_2_ and/or CH_4_ (mL_*n*_/min)	plasma power (W)
pretreatment	1800	40	0	30
cooldown	1800	100	0	0
adsorption	800	20	20	0
flushing	1000	100	0	0
desorption	800	40	0	30
cooldown	1800	100	0	0

Fresh sorbent material was pretreated with plasma to remove any ambient H_2_O or CO_2_ from the surface. In the adsorption stage, a mixture of the adsorption components was fed to the reactor until the sorbent material was saturated. To clear the lines and remove any nonadsorbed CO_2_ and CH_4_, the reactor was flushed with a high flow of Ar. This also ensures that we only measured surface desorption and conversion in the desorption step. Finally, in the desorption step, the plasma was switched on to induce desorption. We applied a frequency of 45 kHz and a constant plasma power of ca. 30 W. More details on the plasma power are presented in the SI, Section S3. After each plasma treatment, the reactor was flushed with Ar for 1800 s to cool down to below 40 °C (measured with a thermal camera on the reactor). Since the adsorption capacity of zeolites decreases due to plasma heating, this approach was needed to maintain consistency in the amount of CO_2_ adsorbed in all tests.

For calculating the adsorbed and desorbed volumes, the total volumetric flow rate is based on the flow rate of Ar, which is set at a constant input value and assumed to be inert, and the molar concentration of Ar. The component-specific flow rate is determined by multiplying the total flow rate with the molar fraction of interest. The total amount of adsorbed and desorbed components was calculated from the integration of the differential concentration over time and corrected for the blank measurement. For the total volume, the integration was made over the entire desorption period (50–450 s), while for the instantaneous volume, the integration was made over the period between two measurements, which is 10 s. These values were averaged over the three repeated experiments to determine the experimental error. While TGA is the conventional method to determine the CO_2_ capacity in material science, we want to stay consistent with the plasma tests, to improve our understanding of the plasma process. Since the adsorption capacity can be influenced by plasma exposure, the capacity calculated via the plasma tests is more reliable, especially since we calculate the average value over three runs.

We can estimate CO_2_ conversion based on the production of CO, similar to previous work on plasma-based CO_2_ splitting^17,40^:
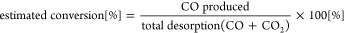
1

It should be noted that the estimated conversion in this case is not the actual representation of the conversion, but a rough estimation to evaluate the chemical process. In some experiments, CH_4_ also plays a role in the plasma and value-added products can be formed. Furthermore, significant carbon deposition on the packing and some condensation at the outlet were observed, making it unfeasible to complete the carbon and hydrogen balance. For example, the measured CO may be formed not only from CO_2_ conversion but also possibly from the oxidation of carbon at the surface.^41^ In addition, the measured concentrations are time-dependent in this nonsteady-state process, which means that the typical calculations and correction for the gas expansion, such as those described by Wanten et al.^40^ for flow plasma reactors, are invalid in our case. This is especially difficult for calculations regarding energy efficiency and cost: due to the significant desorption in these experiments, the variable flow rate is not suitable to determine the specific energy input. Alternatively, we calculate the energy yield based on the duration of the desorption peak and the production of CO:
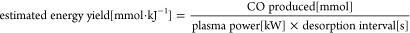
2

The purpose of using Ar during the desorption stage is to use it as a carrier gas to create a flow that enables the measurement of transient concentrations, to study the time-dependent behavior of the plasma-sorbent system. For practical application, a different low-cost carrier gas would be more interesting. It should be noted that switching the discharge gas will alter the plasma properties and a detailed investigation of the effect on the desorption procedure would be needed. A discussion on a more realistic process is given in the Outlook, Section 4.

## 3 Results and Discussion

### 3.1 Operation with CO_2_/CH_4_ Feed Gas

In our first set of experiments, we applied a 1/1/1 CO_2_/CH_4_/Ar mixture as the feed gas in the plasma–sorbent system, with Ar as the internal standard. The experiment was performed according to the general outline described in Table 1 and is presented in more detail in the SI, Section S4. Notably, the pretreatment and cooldown steps remain consistent across all experiments and are thus not explicitly described here but presented in the SI, Section S5.

#### 3.1.1 Adsorption and Flushing

**Figure 2:**
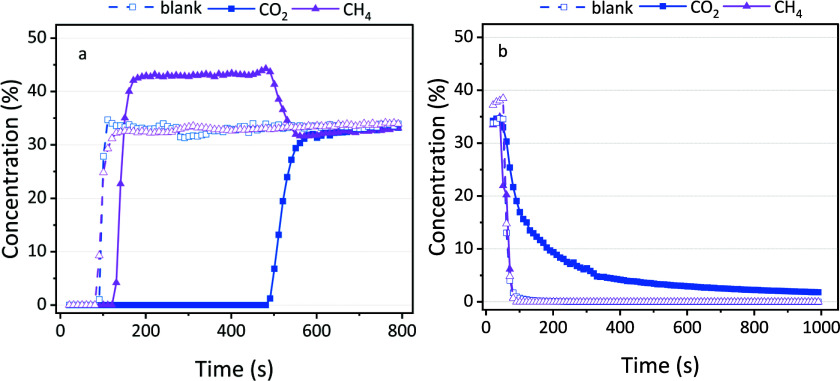
Concentration of CO_2_ and CH_4_ in the outlet stream (a) during the adsorption stage and (b) during the flushing stage. The solid points are for the zeolite, while the open symbols are for the blank measurements with quartz.

Figure 2a displays an example of concentration measured as a function of time for each component during the adsorption step. The open symbols represent the blank measurement over quartz sand, which reveals a measurement delay of approximately 100 s. Since this material does not adsorb either CO_2_ or CH_4_, this delay primarily arises from the time required for the gas to travel through the pipeline and reach the gas cell of the FTIR for measurement.

The blank material has a simultaneous breakthrough of CO_2_ and CH_4_ reaching about 33%, as expected since no gas is adsorbed, and the feed gas has a 1/1/1 CO_2_/CH_4_/Ar ratio. In contrast, for sorbent material zeolite 5A, we observe a clear difference between CO_2_ and CH_4_. The breakthrough of CH_4_ is very fast, and only slightly later than for quartz sand, indicating that no significant amount of CH_4_ adsorbs on zeolite 5A. This is also confirmed by the results obtained in the flushing and desorption steps, as described in the following sections. The small delay in the CH_4_ concentration compared to the blank can be attributed to the reduced total flow rate due to CO_2_ adsorption, which slightly increases the residence time in the gas lines between the reactor and the FTIR. Likewise, the higher concentration (about 45%) compared to the blank is also attributed to CO_2_ adsorption, as there is now relatively more CH_4_ in the mixture. For CO_2_, the breakthrough occurs at a much later time, indicating significant adsorption until the material is saturated after 500 s. Based on three repeated experiments, we calculate an adsorbed volume of 126 ± 1 mL, corresponding to an adsorption capacity of 2.4 mmol/g in line with the literature on zeolite 5A.^42^ After the sorbent is saturated, CO_2_ remains in the gas stream, explaining the rise in its concentration and leading to a decrease in the relative concentration of CH_4_ to a value of about 33%, similar to the blank experiment.

The difference between CO_2_ and CH_4_ in Figure 2a demonstrates a preferential interaction of zeolite 5A with CO_2_ instead of CH_4_, as proven in specific literature on zeolite 5A^42^ and similar materials.^43,44^ For gas mixtures such as biogas, CO_2_ can selectively adsorb on zeolite 5A to give a purer CH_4_ outlet stream. This can be explained by the material properties and the polarizability of the molecules. The quadrupole moment of CO_2_ can induce electrostatic interactions with the cations in the zeolite (Ca^2+^ and Na^+^).^45^ CH_4_ has no quadrupole moment,^45^ and only weaker induction interactions are possible with the zeolite due to the polarizability of the molecule. Some materials might be more suitable for CH_4_ capture,^46,47^ but the competition with CO_2_ is usually not accounted for. In our study, we selected zeolite 5A due to its suitability for the outlined objectives (Section 2.1), and we focused on establishing the proof of concept for sorption-enhanced DRM. Further screening and optimization of sorbent materials is recommended for future study.

Note that the CO_2_ and CH_4_ concentrations and flow rates chosen for this study are based on experimental constraints and the capabilities of the setup. In real-world scenarios, concentrations vary widely depending on the source, from high levels in biogas^31^ and landfill gas^32^ to very low concentrations in flue gas or air. Additionally, zeolite sorbents, like the one in this study, are also investigated for DAC.^48^ Given that only CO_2_ is adsorbed, the CO_2_/CH_4_ ratio is less influential as it only affects the adsorption duration. Thus, the conditions selected for this study were chosen to ensure a reasonable duration for experimentation.

The flushing step removes any residual gas to ensure that the following desorption step is a true representation of only the adsorbed molecules. Figure 2b illustrates the concentration of CO_2_ and CH_4_ during flushing as a function of time. In the blank experiments, after the initial delay due to the gas lines, the concentration drops immediately for both gas components. The same behavior is observed for the concentration of CH_4_ in the case of zeolite 5A. The reduction in CO_2_ concentration occurs slowly, suggesting a gradual release of CO_2_ from the sorbent. The flushed volume of CO_2_ is calculated as 53 ± 2 mL, which is about 40% of the total adsorbed volume. Due to its quadrupole moment, CO_2_ will mostly interact through physisorption with the zeolite, as previously explained. This interaction is notably weaker compared to chemisorption or stronger dipole interactions. The gradual decline depicted in Figure 2b indicates that a significant amount of weakly adsorbed CO_2_ is flushed away from the material, alongside the gas trapped between the pellets. For this proof of concept in this work, this relatively long flushing step was chosen to eliminate the influence of gas residue in the reactor and pipeline, in order to determine the real desorption induced by plasma in the next step. For realistic applications, it is crucial to design shorter flushing steps while also ensuring that weakly adsorbed CO_2_ can be effectively treated by plasma for conversion.

#### 3.1.2 Desorption and the Influence of Temperature

**Figure 3:**
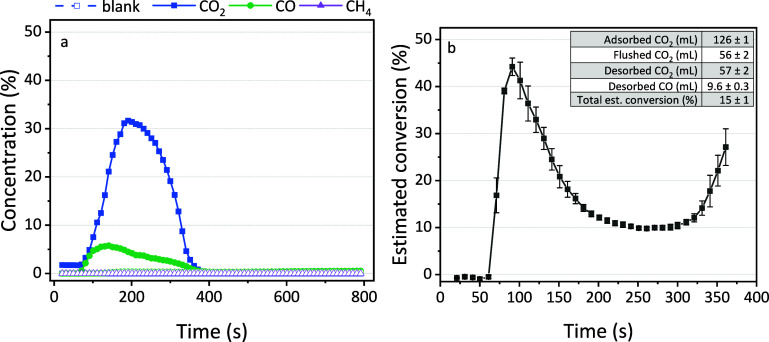
(a) Concentration of CO_2_, CO, and CH_4_ in the outlet stream during the plasma treatment. The solid points are for the zeolite, while the open symbols are for the blank measurements with quartz. (b) Estimated conversion at every time interval during CO_2_ desorption. The values after 360 s are not accurate because the areas are too small; the estimated conversion will drop to zero in reality. The inset summarizes the volumes and total conversion.

The plasma is ignited in Ar with a power of ca. 30 W to induce desorption and conversion. Figure 3a displays the concentrations measured in the outlet stream as a function of time during the desorption.

CO_2_ desorption from the material is evident, while no CH_4_ desorption is detected, consistent with earlier observations of minimal CH_4_ adsorption on the zeolite. While DRM requires both CO_2_ and CH_4_, noticeable adsorption and subsequent desorption are observed exclusively for CO_2_. Nevertheless, CO was detected in the outlet gas stream, indicating that CO_2_ splitting occurs during plasma exposure since typically only CO and O_2_ are formed in DBD plasmas in CO_2_.^49^ The mechanisms for CO_2_ splitting during desorption were discussed in a previous work by Li et al.,^22^ where two routes were suggested: the adsorbed CO_2_ can desorb and convert to CO in the gas phase, or the adsorbed CO_2_ can be split directly and produce gas-phase CO. Even though a different sorbent material was tested in this work, the same mechanisms probably play a role here.

By integrating the area under the curve, we calculated a desorbed volume of 57 ± 2 mL for CO_2_ and a volume of 9.6 ± 0.3 mL for CO. In addition to the flushed volume of 56 ± 2 mL in Figure 2b, we obtained a total volume of 120 ± 3 mL that was removed from the sorbent. This corresponds to 95% of the total adsorbed volume of CO_2_ (126 ± 1 mL; Figure 2a). The 5% difference may be caused by the resolution of the FTIR. Although the measurements are taken every 10 s, it still could introduce an error on the steep gradients in our results. Despite this small deviation, we can determine the estimated conversion as 15 ± 1% according to eq 1, using the total areas of the CO_2_ and CO peaks. The maximum estimated transient conversion, calculated for each point, is 44 ± 2% obtained around 91 s (see Figure 3b). The highest value is expected at the beginning of the desorption peak since CO_2_ is heavily diluted in Ar. It is well known for CO_2_ conversion in DBD plasma that dilution in Ar can improve the conversion.^50^ Notably, a rise in CO_2_ concentration correlates with a decreasing conversion, and vice versa, indicating a direct inverse relationship between these two parameters. Still, both estimated conversions are in the range of typical values obtained in DBD plasmas.^52^ The estimated energy yield is 0.047 mmol kJ^–1^ (see eq 2). A desorption time of 300 s was chosen because this was the time of most significant conversion. This is significantly lower than the energy yield obtained in CO_2_ conversion in flow DBD reactors. In the work by Wang et al.,^51^ where a zeolite 5A packing similar to this work was used, they had a similar CO_2_ conversion 15% but a much better energy yield of 33.3 mmol kJ^–1^. However, we cannot directly make this comparison with the energy efficiency and cost in steady-state flow reactors (as discussed in Section 2.2). It should be mentioned that the aim of this work was solely to demonstrate the proof of concept. Further optimization to improve performance can be achieved through enhanced sorbent capacity, better reactor design, and refined operation parameters, which will be investigated in the future.

**Figure 4:**
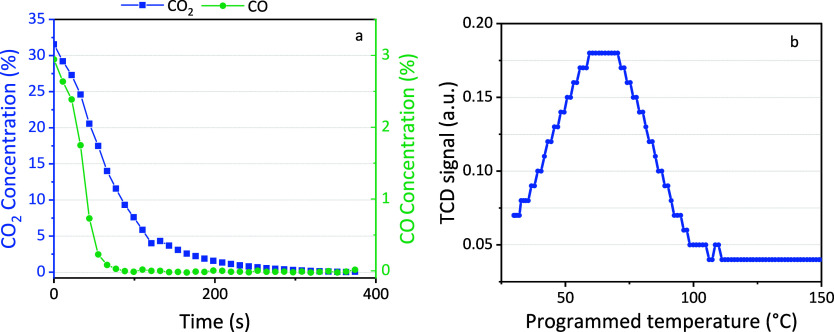
(a) Concentration of CO_2_ and CO (left and right *y*-axis, respectively) as a function of time after the plasma is turned off (treatment time of 200 s of plasma desorption). (b) Temperature-programmed desorption (TPD) of zeolite 5A with the thermal conductivity detector (TCD) signal in arbitrary units, representing the CO_2_ desorption. The step size of the *y*-axis was set to 0.01, explaining the step-like profile of the curve.

To verify the desorption and reaction mechanism in our system with the zeolite sorbent, we ran the plasma-desorption step for 200 s and then we switched off the plasma during desorption and started the measurement. Figure 4a depicts the CO_2_ and CO concentrations as a function of time, starting when the plasma was switched off. The CO concentration drops abruptly, proving that there is no conversion without plasma. However, the CO_2_ concentration decreases more gradually when the plasma is switched off. Although CO_2_ has a higher initial concentration, which could influence the decrease rate, in our experimental system, the continuous Ar flush at 40 mL_*n*_/min could decrease the CO_2_ concentration to <1% within 100 s if no further desorption occurred. Instead, we observed a sustained CO_2_ concentration >1% until 220 s. This indicates that the desorption does not stop instantly, as in the case of CO. The sustained desorption is primarily attributed to the thermal effect, resulting from the plasma heating of the sorbent.

Temperature-programmed desorption (TPD) was conducted to study the thermal desorption profile, and the result is presented in Figure 4b. CO_2_ desorbs from zeolite 5A at temperatures between 40 and 100 °C. Even though DBD generates a relatively cold plasma, the temperature increase of the sorbent could easily reach the level needed for desorption on zeolite 5A.^49,53^ Especially the localized plasma heating, which is widely acknowledged as the so-called “hot spots”,^54^ could play an important role. Direct measurement of the sorbent surface temperature is not feasible in our case. Instead, we installed an IR camera to estimate the temperature of the reactor wall during plasma operation as an indication.

**Figure 5:**
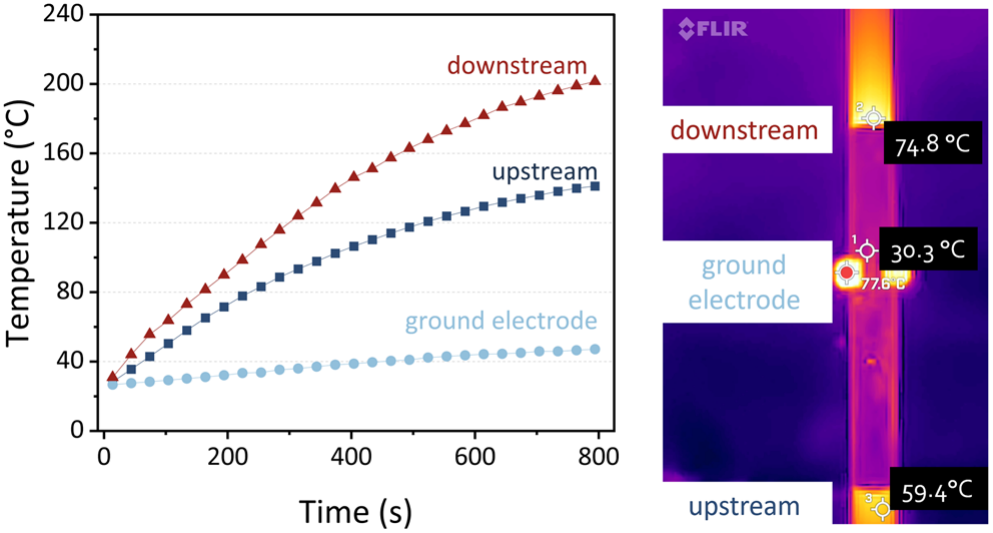
Temperature at the outside of the DBD reactor packed with zeolite 5A during plasma operation (left) and the thermal image (right), taken at 140 s. The grounded electrode remains dark in the image because the metal has a low emissivity (0.1–0.15) compared to the ceramic (0.95); hence, the IR camera cannot measure the temperature appropriately.

The results are presented in Figure 5, and an approximate comparison with TPD-MS is presented in the SI, Section S6. It is important to note that the sorbent temperature inside the reactor could be even higher, as we only measured the temperature after heat transfer through the wall. Furthermore, the IR camera identified the hottest point on the image. In this snapshot, the value is 77.6 °C (at the steel clamp attachment of the ground electrode), but since this point is variable over time, it was not included in the left graph. The steel clamp was only the hottest initially, due to Ohmic heating of the current flow and due to heat transfer from the reactor body. This is more visible in the steel clamp than in the aluminum ground electrode due to the low emissivity of aluminum. Later, the downstream area became warmer and the maximum temperature point shifted.

Both the upstream and downstream reactor walls heat up very quickly. Within 200 s, the temperature is higher than 60 °C, which can induce desorption according to the TPD (cf. Figure 4b). The temperature of the wall continuously increases up to 200 °C. During the cooldown period, the reactor wall temperature also remains above 80 °C within 400 s after switching off the plasma. This can explain the sustained desorption that we observed in Figure 4a and indicates that the temperature plays an important role in the desorption, although other mechanisms cannot be ruled out. Possibly, reactive plasma species, such as electrons, ions, radicals, and excited molecules produced in the plasma, can interact with the surface and also enhance the desorption, as discussed in the work by Yoshida et al.^21^ where plasma desorption was much quicker than thermal desorption. However, these temperatures of DBD plasma are not enough for thermal CO_2_ splitting,^18^ as demonstrated by the sudden drop in CO concentration in Figure 4a. The effect of plasma is twofold: (1) heating the material to induce desorption and (2) splitting the desorbed CO_2_ into CO. This combination is crucial to achieving real carbon utilization in the plasma–sorbent system.

### 3.2 CO_2_ Adsorption Followed by Desorption in Ar/CH_4_ Plasma

**Figure 6:**
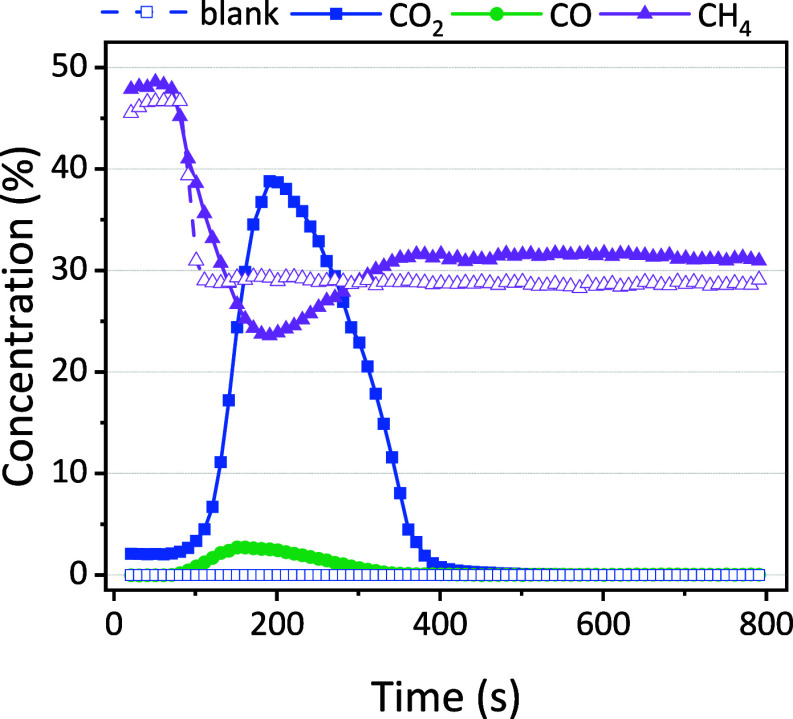
Concentration of CO_2_, CO, and CH_4_ in the outlet stream during the desorption stage when the Ar/CH_4_ plasma is ignited. The solid points are for the zeolite, while the open symbols are for the blank measurements with quartz.

As discussed in the previous section, only CO_2_ adsorbs significantly, meaning that sorbent-based DRM is not feasible with a CO_2_/CH_4_ mixture as the feed gas. Instead, an alternative approach can be implemented by introducing CH_4_ to the carrier gas during the desorption stage with plasma. To validate this, CO_2_-saturated sorbents were flushed with a CH_4_/Ar mixture and subsequently exposed to plasma in the same CH_4_/Ar mixture. Detailed experimental procedures can be found in the SI, Sections S4 and S7. Figure 6 presents the concentrations as a function of time during the desorption step when the Ar/CH_4_ plasma is ignited.

The concentrations of CO_2_ and CO exhibit similar profiles as in the previous section, indicating that the CH_4_/Ar plasma is also suitable to desorb CO_2_ and convert it to CO. The concentration of CH_4_ drops within the initial 200 s, due to three primary factors: (1) CH_4_ is consumed via nonoxidative coupling into higher hydrocarbons induced by the plasma, which also explains the drop observed in CH_4_ concentration in the blank experiments; (2) dilution of the relative concentration due to CO_2_ desorption from the sorbent into the gas phase; (3) CH_4_ consumption as a result of reactions with desorbed CO_2_. Due to the latter two reasons, the CH_4_ concentration decreases to a lower level in the case of zeolite 5A compared to the blank experiment. Subsequently, it begins to increase and reaches a plateau, corresponding to the declining CO_2_ desorption over time.

Interestingly, there is a small difference between the CH_4_ concentrations of the blank and the zeolite after 500 s, even though the desorption is finished. The small deviation might be attributed to the water formation in the experiment with the zeolite sorbent, which can bind to the zeolite and influence the reaction, as discussed in more detail in Section 3.2.2. Indeed, even small amounts of H_2_O can influence the reaction,^55^ which can explain this difference.

The estimated conversion based on the CO production is about 7 ± 1%, with an estimated energy yield of 0.0188 mmol kJ^–1^. This is significantly lower than the estimated conversion of 15 ± 1% in Section 3.1.2 because other products such as water can be formed due to the CH_4_ addition. Indeed, DRM should primarily occur during CO_2_ desorption, i.e., between 100 and 400 s, and pure CH_4_ conversion (i.e., nonoxidative coupling) takes place after 400 s. Although the measured spectra suggest the formation of C_2_H_6_ and C_2_H_2_ (SI, Section S8), quantification remains challenging due to their spectral overlap with CH_4_ peaks. Therefore, we conducted additional GC measurements, supplementary to the FTIR measurement, for better analysis of products and reactions.

#### 3.2.1 Product Verification

**Figure 7:**
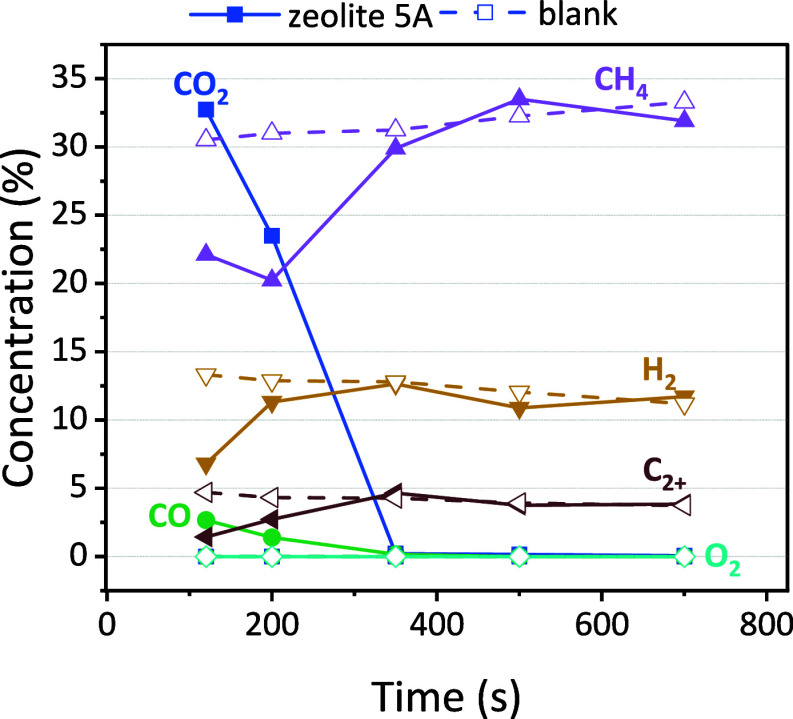
Concentration of all different components identified by GC for discrete points in time. The solid points are for the zeolite, while the open symbols are for the blank measurements with quartz.

The results of the GC measurements are depicted in Figure 7. Due to limitations in the setup, we could only measure discrete points in time, as discussed in Section 2.1.

Not only H_2_ is formed but also C_2+_ hydrocarbons, including C_2_H_6_, C_2_H_4_, C_2_H_2_, and C_3+_ products. Some CO_2_ and CO are detected in the beginning at 100 and 200 s, similar to the desorption peak that we measured in Figure 6. For the blank material, there is no CO_2_ and CO detected, corresponding to the fact that no adsorption of CO_2_ occurred. As expected, the results with the sorbents are the same as the blank measurement after about 500 s. This is because the desorption of CO_2_ is finished (Figure 6) and both materials display simply nonoxidative coupling of the CH_4_ plasma. The results might be different if a catalyst would be included on the zeolite and could possibly alter the selectivity, which will be part of future work.

From this figure, it is difficult to evaluate whether the desorbed CO_2_ and CH_4_ effectively interact during the possible DRM period (100–400 s). However, two observations do indicate the occurrence of DRM in our system. First, throughout the tests, no O_2_ was detected, potentially due to the formation of H_2_O and CO, which consume O atoms generated in the plasma from CO_2_.^56^ Second, condensation observed at the reactor outlet suggests the formation of liquid products, possibly including some oxygenated compounds, but primarily expected to be H_2_O, based on prior research in DBD plasma.^57,58^ To further explore the role of H_2_O as an indicator for DRM, the relative humidity of the outlet gas stream was monitored with a humidity meter.

#### 3.2.2 The Role of H_2_O

**Figure 8:**
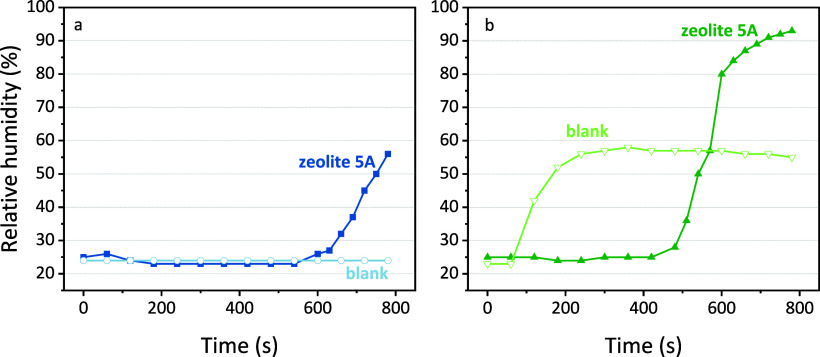
Relative humidity as a function of time for (a) the desorption procedure where the CH_4_/Ar plasma is ignited on the zeolite with adsorbed CO_2_ from a previous step and (b) a typical flow plasma reaction with CO_2_ and CH_4_ as the feed gas without previous adsorption steps on the zeolite packing material.

The results of the relative humidity in the outlet gas stream are shown in Figure 8. The measured H_2_O is an indicator for DRM; however, it is also important to understand the plasma–sorbent interaction. Note that the base humidity is not equal to zero, but since it remains consistent at about 25% after sufficiently flushing the system with dry input gas, the results of this experiment were deemed appropriate to understand different trends. The timing of 800 s was chosen to have an exact comparison to the desorption step in Figure 6.

First, we determined the humidity in the desorption procedure, where the time corresponds to the start of the desorption stage in which plasma is ignited and the desorption of CO_2_ takes place. In Figure 8a, the humidity suddenly increases after 600 s for zeolite 5A. This indicates that the desorbed CO_2_ is sufficient to react with CH_4_ in the gas phase and confirms that the procedure in this work enables sorption-enhanced DRM, with H_2_O as a byproduct. For the blank measurement, this is not the case, since there is no desorbed CO_2_ that could interact with CH_4_ to form H_2_O, and the humidity stays constant. In addition to the desorption-based DRM, we also performed a typical “flow” experiment, where both CO_2_ and CH_4_ were simply used as feed gases during the plasma experiment in a 1/1 ratio and without any previous adsorption step, hence classical plasma-based DRM. The detailed results of this flow experiment are presented in the SI, Section S9. In Figure 8b, the humidity when using quartz sand packing rises rapidly, indicating that H_2_O is formed quickly in the blank experiments. For the zeolite 5A, however, there is a significant delay in the detection of H_2_O. Indeed, zeolite 5A is known to bind H_2_O very strongly thanks to its dipole.^45^ Observations of the outlet confirm the results of the humidity meter. When a higher humidity was measured, some condensation also formed in the outlet of the reactor, although there was not enough liquid for quantification.

This result could open the path to new applications in gas conversion with plasma technology. In DRM, H_2_O is usually an unwanted byproduct in the outlet stream.^59^ The zeolite material is able to capture the formed H_2_O for *in situ* product removal, which can shift the equilibrium and enhance the conversion in plasma processes.^60−62^ One could design a type of chemical looping process to exploit these properties, similar to catalysts.^63^ For example, in the first stage, the DRM reaction can continue for about 450 s (or as long as CO_2_ desorption continues) with an H_2_O-free product stream. In the second stage, before humidity increases, the feed gas can be switched to a carrier gas (such as N_2_ and Ar) for a plasma treatment, to remove the H_2_O and recover the sorbent material. Indeed, although most gas conversion research in typical flow plasma reactors aims for steady-state operation, these insights on shorter time scales provide a promising alternative.

### 3.3 Influence of Power during Desorption

**Figure 9:**
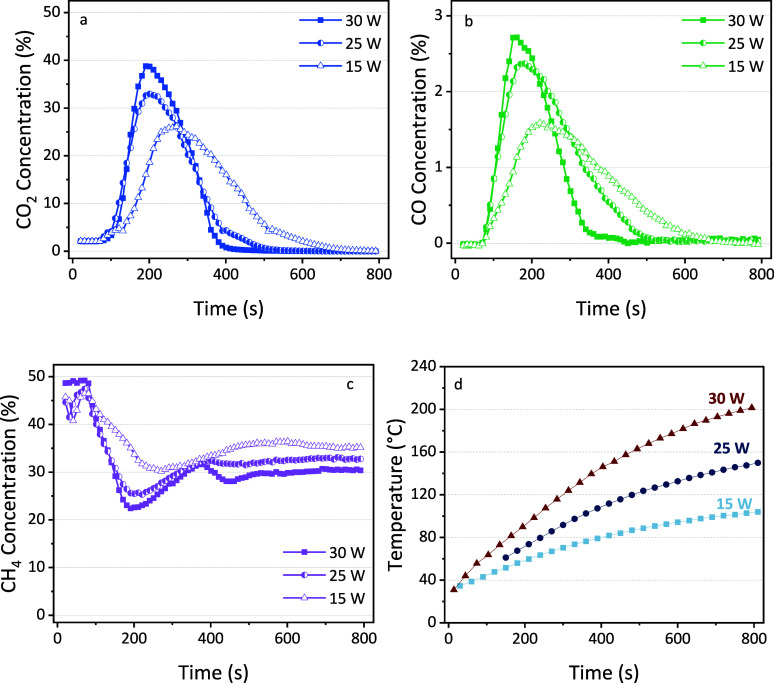
Concentration of CO_2_ (a), CO (b), and CH_4_ (c) as a function of time for different plasma powers. For clarity, there are no blank measurements displayed here. The downstream temperature of the reactor wall measured with a thermal camera is given in (d).

To investigate the influence of discharge power, experiments were conducted using the same procedure at power levels of 25.1 ± 1 W (ca. 25 W) and 16.3 ± 1.5 W (ca. 15 W). The results are shown in Figure 9, alongside the previous results obtained from experiments conducted at 30 W. In all experiments, the same cooldown interval of 1800 s was maintained.

The concentration peaks of both CO_2_ and CO shift to later times when lower power is applied, and we observe sharper peaks with a higher maximum in the case of ca. 30 W. These observations align with findings reported in a previous work.^17^ The shift to later times (and a lower maximum) is attributed to fewer active species generated by the plasma and a more moderate heating effect at lower power, as indicated by the reactor wall temperature displayed in Figure 9d. Simultaneously, at lower plasma power, the measured concentration of CH_4_ is higher. This results from the reduced CH_4_ consumption at lower power.

**Figure 10:**
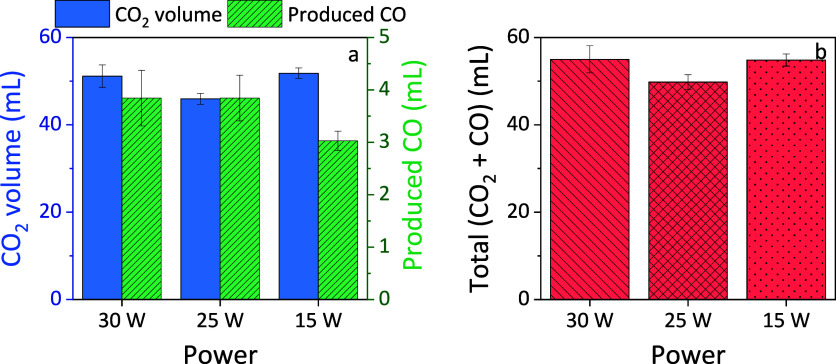
(a) Desorbed amount of CO_2_ and measured CO and (b) the sum of CO_2_ and CO for the three different powers tested in this work.

Additionally, the total volumes of CO_2_ and CO as well as the sum of both are shown in Figure 10. It can be observed that the overall sum of CO_2_ and CO produced under different power settings is generally similar, with minor variations noted. There was no significant difference observed between the cases of 30 and 25 W. However, the production of CO was lower at 15 W. This discrepancy can be attributed to a combination effect of several factors. First, lower power led to slower desorption of CO_2_ into the gas phase, affecting its conversion via gas-phase reactions (as shown in Figure 9). Additionally, plasma with lower power results in less energy input, meaning that fewer reactive species are generated to facilitate the conversion reaction. Compared to a reactor operating with constant reactant input (continuous feed gas flow of reactant), the plasma–sorbent system is inherently more complex. The observed combination effect cannot be adequately explained by one or two factors alone. Therefore, further in-depth investigation is necessary to fully understand the underlying mechanisms at play.

### 3.4 Material Analysis

As known in plasma catalysis and from previous work, exposure to plasma can change the physical properties of a packing material.^17,54^ To investigate the effect of the different operating powers and the duration of the plasma treatment on the material, we measured the BET surface area and BHJ pore volume. The results are summarized in Table 2, and these values are in line with the range reported in the literature.^64,65^

**Table 2:** Surface Area and Pore Volume of the Untreated Zeolite 5A Sorbent, Compared to the Different Procedures for Plasma Desorption^a^

	BET surface area (m^2^·g^–1^)	BHJ pore volume (cm^3^·g^–1^)		BET surface area (m^2^·g^–1^)	BHJ pore volume (cm^3^·g^–1^)
untreated	577	0.242	**untreated**	577	0.242
30 W	534	0.218	**1 run**	544	0.224
25 W	536	0.219	**3 repeats**	534	0.218
15 W	546	0.224	**6 repeats**	523	0.212

a The benchmark of Section 3.2 is the desorption at ca. 30 W in 3 repeats, underlined in the table.

The surface area and pore volume decrease when comparing the fresh sample to the treated samples. The carbon deposition from CH_4_ plasma, which we could observe visually on the sample, plays a key role by clogging the porous structure. Pictures from the samples, as well as the results from a thermogravimetric analysis (TGA) are presented in the SI, Section S10.

**Figure 11:**
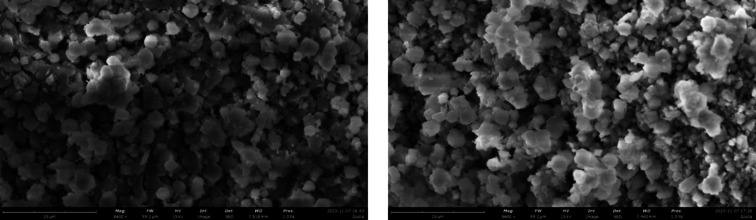
SEM images of the untreated zeolite (left) and the zeolite after six adsorption/desorption repeats (right).

The difference between the different powers is less significant. There is a minor increase in both the BET surface area and the BHJ pore volume when decreasing the power from ca. 30 W in the basic procedure to ca. 15 W. Furthermore, when we compare results for a single run, three repeats, and six consecutive repeats, they demonstrate a decrease in surface area and pore volume, which might inhibit long-term performance. Additional cleaning steps with for example O_2_ plasma, might overcome this issue. Nevertheless, we observed no significant morphology change after six runs compared to the untreated sample, as indicated by the SEM images (Figure 11). Although some morphological changes might be too localized to capture with this SEM resolution, the parameters in Table 2 indicate that the overall effect will be very small.

## 4 Conclusions and Outlook

In this work, a plasma–sorbent system for reactions of CO_2_ and CH_4_ was investigated. When using a CO_2_/CH_4_ input mixture, zeolite 5A selectively absorbed CO_2_ and acted as a filter to produce a purer CH_4_ outlet stream. During the subsequent desorption step with Ar plasma, only CO_2_ was desorbed from the material. We demonstrated the significant impact of plasma heating on the desorption process. During plasma operation, the reactor temperature could readily attain the levels necessary for thermal desorption of CO_2_ from zeolite 5A. Furthermore, the production of CO arises from plasma-induced reactions and can be controlled instantly by switching the plasma on/off.

DRM from adsorbed components proved unfeasible via direct feeding of a CO_2_/CH_4_ mixture since only CO_2_ demonstrated significant adsorption and desorption. Instead, we explored an alternative approach, involving CH_4_ addition during plasma-induced desorption of preadsorbed CO_2_. This yielded an output stream containing various value-added chemicals, including H_2_, CO, C_2_H_6_, C_2_H_4_, C_2_H_2_, and C_3+_. Although most of these products (except CO) could also be formed by CH_4_ nonoxidative coupling only, the presence of H_2_O suggested possible DRM reactions. Notably, zeolite 5A showed potential for *in situ* removal of H_2_O, shifting the equilibrium and possibly enhancing the conversion in the plasma. Furthermore, the surface area and pore volume of zeolite 5A decreased after plasma exposure, caused by the carbon deposition on the sorbent. However, the material remained stable since we observed no significant morphological changes.

Overall, the plasma–sorbent system in this work provided an interesting proof of concept, but some remaining challenges require further investigation. The sorbent material could be tailored for a higher adsorption capacity and stability during gas conversion, as is already the subject in the field of material science for dual functional materials.^66,67^ The possible competition between CO_2_ and H_2_O adsorption could be further investigated. An ideal material for this process would be stable in the plasma discharge, resistant to carbon deposition, with a high adsorption capacity, and should adsorb both CO_2_ and CH_4_. Alternatively, a mix of materials for either CO_2_ or CH_4_ adsorption could be more feasible. It should be noted that the field of CH_4_ adsorption remains challenging, especially in competition with CO_2_, but some specifically tailored zeolites show promise.^47,68^ Another modification would be to include a catalyst material on the sorbent, as studied in the field of plasma catalysis^19^; this could yield the production of different value-added chemicals. However, the influence on the adsorption–desorption mechanisms is an additional parameter to be considered.

Furthermore, the procedure for adsorption–flushing–desorption could be tuned for better performance, even at the lab scale. This includes optimization of the flow rate and time for each step. For instance, employing short or moderate flushing can retain weakly adsorbed CO_2_, which can then be utilized in later stages. Moreover, in this study, Ar was used as the carrier gas for accurate measurement of transient concentrations, but this is not necessary for practical applications considering the additional cost. Exploring cost-effective carrier gases like N_2_ is needed, or even batch reactors can be considered, which can efficiently leverage plasma heating for desorption and offer longer reaction residence times. External heating could be investigated as well to speed up the desorption, although this would increase the energy cost and require careful tuning of the residence time to ensure sufficient conversion.

In addition, exploring innovation at the process level holds potential for a more practical utilization of mixed sources such as biogas. For instance, utilizing sorbents such as zeolite 5A, which selectively adsorb CO_2_ as demonstrated in this study, allows for the initial separation of CH_4_ from the inlet stream during the adsorption step. Subsequently, this CH_4_ can be reintroduced into the reactor during desorption steps (via periodic operation with redirection of the flow or using a multireactor system) to conduct DRM with preadsorbed CO_2_, as demonstrated in our paper. The CO_2_/CH_4_ ratio can be tuned in this way, which will be beneficial, and the conversion can be enhanced due to the adsorption of produced H_2_O, as also observed in our study.

Finally, the plasma reactor itself can also be investigated to enhance its performance. Both reactor design and operating parameters can significantly influence the conversion and energy efficiency, as investigated by many other studies in the field.^69,70^ A multidisciplinary approach for all the abovementioned areas is needed to investigate the promising potential of sorption-enhanced DRM.

## Supporting Information Available

Contains the results from zeolite 4A test, as well as the adsorption capacity, Lissajous figures to describe the plasma power and specific experimental outline of each separate experiment; the pre-treatment results are presented, results from a TPD-MS test, additional figures on CO_2_ adsorption and CH_4_ flushing, detailed FTIR spectra, additional tests of the flow configuration and the results from the TGA (PDF)
