# Supplementary material for: Sorption-Enhanced Dry Reforming of Methane in a DBD Plasma Reactor for Single-Stage Carbon Capture and Utilization
Source: ACS Sustain Chem Eng. 2024 Jul 6;12(29):10841–53. doi: 10.1021/acssuschemeng.4c02502 (PMC11267637; doi:10.1021/acssuschemeng.4c02502)
Supplement: Supplementary file 1 — sc4c02502_si_001.pdf [file sc4c02502_si_001.pdf]

Supporting Information

Sorption-enhanced dry reforming of methane in a DBD plasma reactor for single-stage carbon capture and utilization

Rani Vertongen[1], Giulia De Felice,[2] Huub van den Bogaard,[2] Fausto Gallucci, [2]
Annemie Bogaerts[1] and Sirui Li[2]*

[1] Research group PLASMANT, Department of Chemistry, University of Antwerp,
Universiteitsplein 1, 2610 Antwerp, Belgium.

[2] Research group Inorganic Membranes and Membrane Reactors, Sustainable Process Engineering, Department of Chemical Engineering and Chemistry, Eindhoven University of Technology,
De Rondom 70, Eindhoven 5612 AP, the Netherlands.

*Corresponding author: dr. Sirui Li, email: s.li1@tue.nl

Number of pages: 11

Number of figures: 11

Number of tables: 2

S1. Zeolite 4A

Although a detailed material study was out of scope for this work, we performed some pretests with zeolite 4A. As the results in Figure SI - 1 demonstrate, zeolite 4A has a much lower adsorption capacity, with an early breakthrough curve and small CO_2 volume during desorption. This is in line with previous investigations on adsorbents.[1] The material has a large effect on the performance and leaves room for improvement in future work.

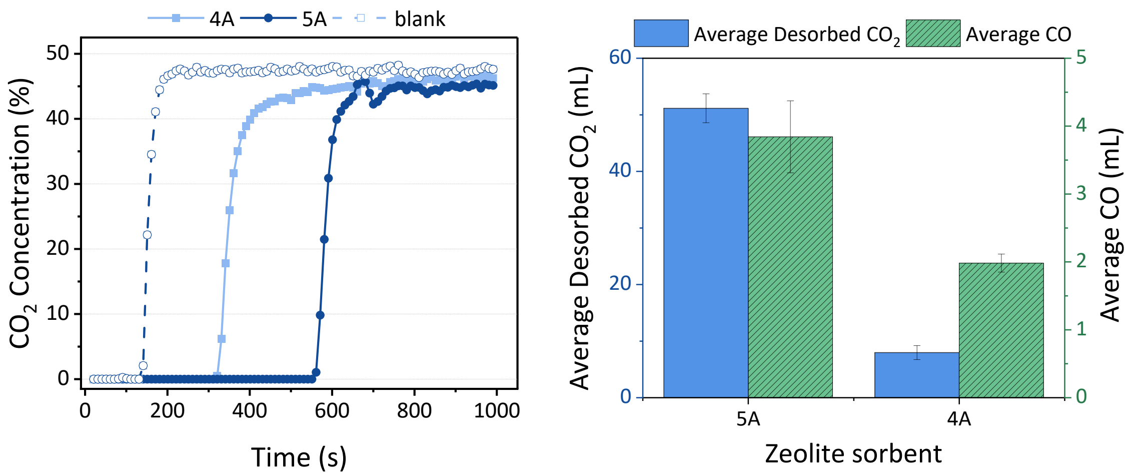

Figure SI - 1 Comparison of zeolite 4A and 5A in terms of (left) CO_2 adsorption as a function of time for a single experiment and (right) CO_2 desorption and CO production averaged over three repeated experiments; note the different scales for CO_2 and CO. The solid points are for the zeolite, while the open symbols are for the blank measurements with quartz.

S2. Adsorption capacity

We repeated each experiment three times on the same powder. To check the viability of this approach, we plot the adsorbed amount of CO_2 for each separate run in Figure SI - 2. These results are obtained with the procedure from Section 3.2 in the main paper, to study the influence of the CH_4 plasma during desorption. The adsorbed volume of CO_2 is slightly higher in the first run compared to the second and third runs (1% decrease), possibly due to carbon deposition (see Section 3.4 in the main paper) or H_2O adsorption (see Section 3.2.2 in the main paper), but the error on the average remains very small. In other words, this approach is suitable for a quantitative comparison between the procedures.

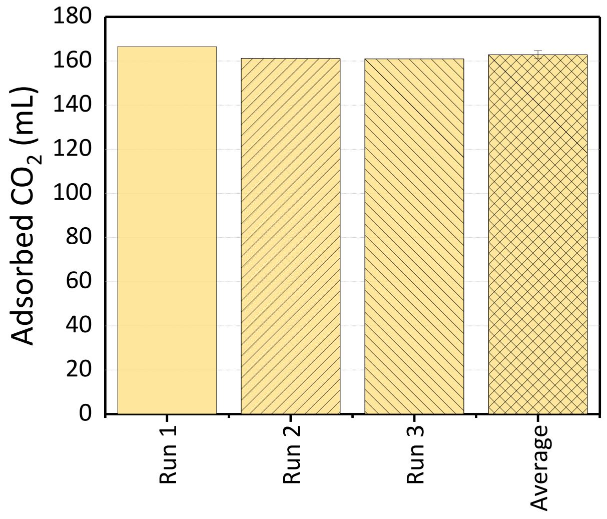

Figure SI - 2 Adsorbed amount of CO_2 for three runs separately and summarized by the average.

S3. Plasma power

A typical Lissajous figure to calculate the power is presented in Figure SI - 3. The plasma power is nearly constant in most experiments, the measured plasma power is on average 31 ± 1.5 W. To simplify the notation, we describe this as "ca. 30 W" in the main paper.

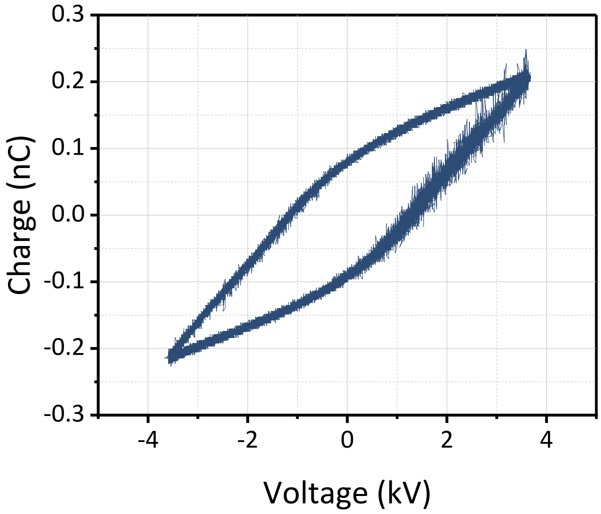

Figure SI - 3 Q-V diagram, also called Lissajous plot,[2] to illustrate the power calculation during plasma-desorption (zeolite 5A packing, 20/20 mL_n/min CH_4/Ar, CO_2 desorption).

S4. Specific experimental outline

The exact outline of each experiment is presented in this Section.

Table SI - 1 Overview of the adsorption-desorption procedure with a mixed CO_2/CH_4 flow during the adsorption stage, as presented in the main paper in Section 3.1.

	Time (s)	Ar (mL_n/min)	CO_2 (mL_n/min)	CH_4 (mL_n/min)	Plasma power (W)
**Adsorption**	800	20	20	20	0
**Flushing**	1000	100	0	0	0
**Desorption**	800	40	0	0	30

Table SI - 2 Overview of the adsorption-desorption procedure with CH_4 addition in the desorption step, as presented in the main paper in Section 3.2.

	Time (s)	Ar (m_nL/min)	CO_2 (mL_n/min)	CH_4 (mL_n/min)	Plasma power (W)
**Adsorption**	800	20	20	0	0
**Flushing**	1000	50	0	50	0
**Desorption**	800	20	0	20	30

S5. Pre-treatment results

We performed the pre-treatment of zeolite 5A utilizing an Ar plasma for 1800 s at a plasma power of ca. 30 W. A typical result is presented in Figure SI - 4. The initial drop in CO_2 concentration is due to the flushing of the lines. At about 100 s, there is a clear desorption peak of CO_2 that was previously adsorbed from the ambient atmosphere and full desorption is achieved at 500s. The small CO peak is the result of CO_2 conversion. Preliminary tests showed that additional drying of the sample for 24h gave the same results as applying the plasma-treatment only.

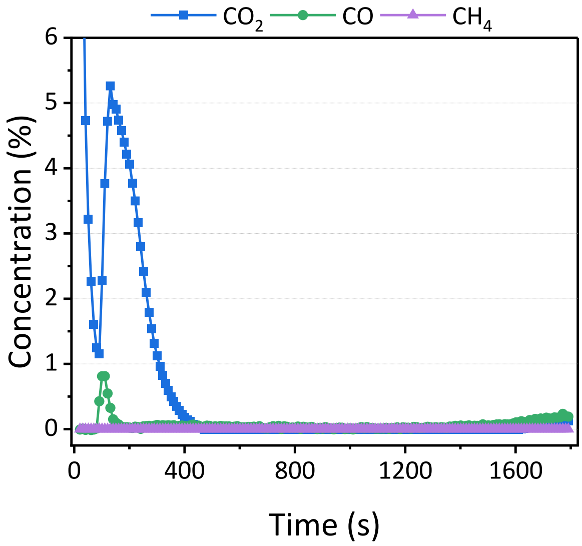

Figure SI - 4 Concentration of CO_2 and CH_4 in the outlet stream during pretreatment.

The concentrations during the cool down phase fall quickly below 0.2%, hence, we do not present these results in a graph.

S6. Approximate comparison with TPD-MS

A direct comparison between the plasma experiment and a thermal test is not possible. The plasma-induced heating is rather complex, since the reactor is heated not through a contact surface inside/outside of the reactor, but by the microdischarges and chemical reactions in the plasma, causing hot spots and a non-uniform heating profile. Still, we made an approximate comparison between the plasma test and a TPD-MS measurement. The procedure was performed according to the specifications in Table 1 in the main paper, but after the flushing stage, the sample was transferred to a sample holder for TPD-MS. A heating rate of 15°C/min was applied for the first 500 s, and then 8°C/min for the time of 500-800 s, to mimic the temperatures in Figure 5. The results are presented in Figure SI - 5.

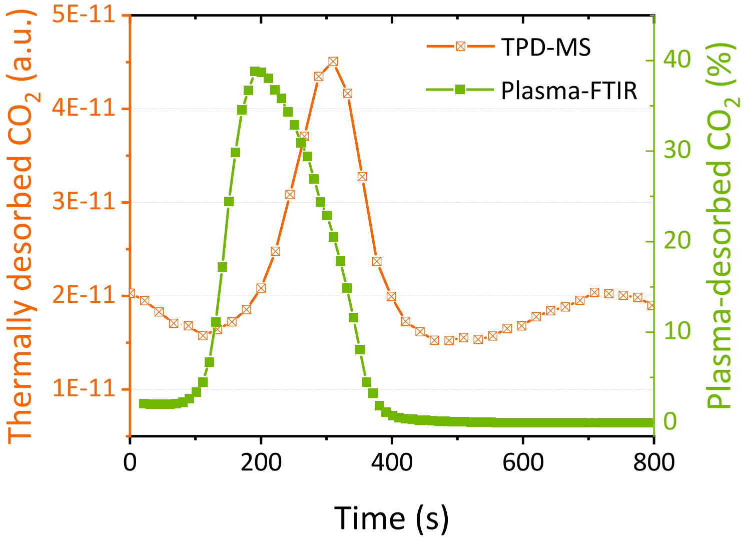

Figure SI - 5 CO_2 concentration measured in an approximate TPD-MS test (left axis) and through the usual plasma-FTIR experiment (right axis). Note that the scales of this figure are not representative, since the MS signal is in arbitrary units.

There is a sharp peak of desorption around 300 s, but the peak in the plasma test is earlier. Since the temperature setting of TPD-MS was chosen based on the temperature measurement of the surface of the plasma reactor (Figure 5 in the main paper), this applied temperature is lower than the actual temperature of the bed, which can explain the late appearance of the peak. Importantly, no CO was measured in the TPD-MS test, confirming that no CO_2 conversion occurs at these temperatures without the plasma.

S7. CO_2 adsorption and CH_4 flushing

Similar to Section 3.1.1 in the main paper for the CO_2/CH_4 mixture, we measured the concentrations during CO_2 adsorption and CH_4 flushing as a function of time with an in-line FTIR, and the results are presented in Figure SI - 6.

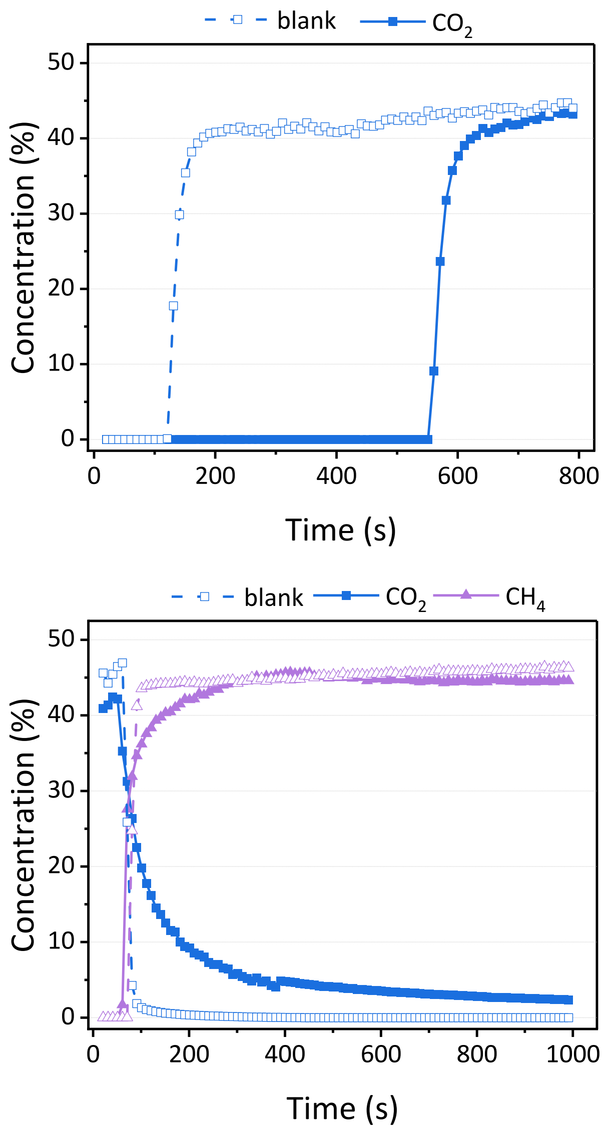

Figure SI - 6 Concentration of CO_2 and CH_4 in the outlet stream during the adsorption (top) and flushing (bottom) stage. The solid points are for the zeolite, while the open symbols are for the blank measurements with quartz.

The adsorption of CO_2 has a breakthrough around 500-600 s, even when no CH_4 is present in the mixture, hence very similar to the experiments in Section 3.1.1 for the CO_2/CH_4 mixture. This is expected because the same flow rate of CO_2 is maintained in both cases due to the lower limit of the mass flow controllers. The CO_2 concentration remains slightly lower than the blank, although it continues to increase slowly after the breakthrough point. This might be related to slow, continued adsorption, but initial tests revealed that this amount is not significant for these experiments.

CH_4 is added during the flushing step to saturate the volume between the pores and eliminate any possible delays on the mass flow controllers during the desorption phase. It has a similar profile over the zeolite as over the quartz material.

Despite the varying composition of the gas during the plasma desorption process, the plasma power remains constant, as shown in Figure SI - 7. The measured plasma power is on average 31.6 ± 0.1 W. The fact that the plasma power remains constant can be expected since the ionization potentials of CO_2 and CH_4 are similar.[3]

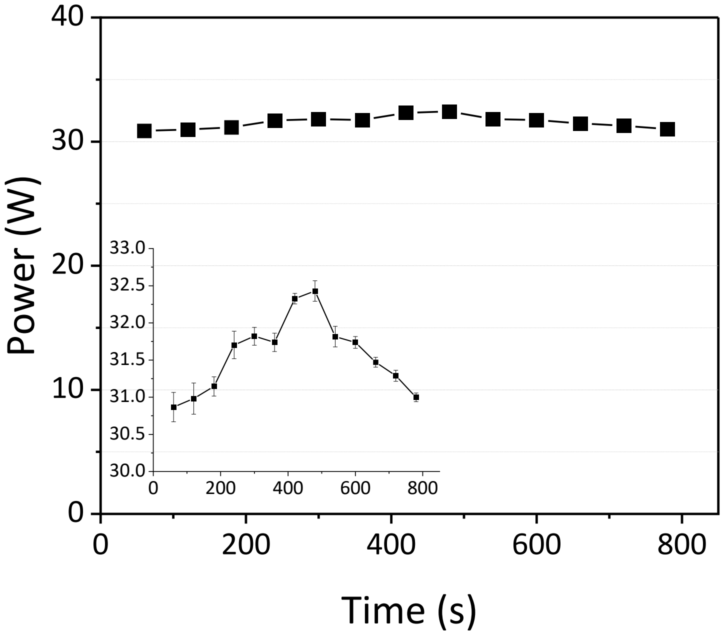

Figure SI - 7 Plasma power as a function of time during the desorption step. The error bars are too small to be visible. The inset presents a finer resolution on the y-axis.

S8. FTIR spectra

A typical FTIR spectrum of an experiment of Section 3.2 in the main paper is presented in Figure SI - 8. The peaks were designated based on the NIST database.[3] At 300 s, there is clear CO_2 desorption and some conversion to CO, but the desorption seems over by 400 s. At both points in time, the production of C_2H_6 is observed, as well as C_2H_2, an indication of methane non-oxidative coupling.[4]

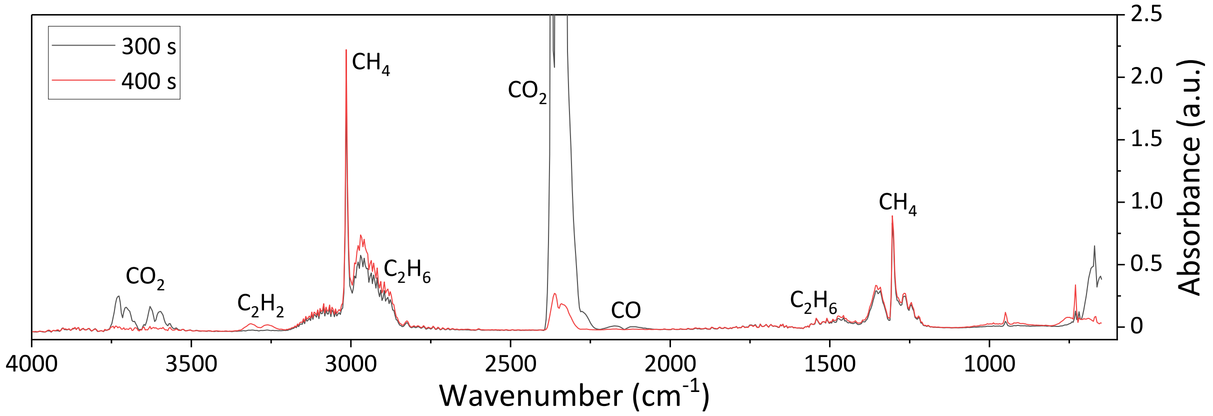

Figure SI - 8 FTIR spectrum of the CH_4 plasma-induced desorption of pre-adsorbed CO_2. Note that the y-axis is presented on a smaller scale to visualize the smaller peaks. In the full resolution, no peaks are saturated in the spectrum.

S9. Sorption vs flow configuration

In order to compare the desorption procedure with the typical “flow” plasma reaction, we performed two experiments with a CH_4 and CO_2/CH_4 flow at a plasma power of ca. 30 W without an adsorption step.

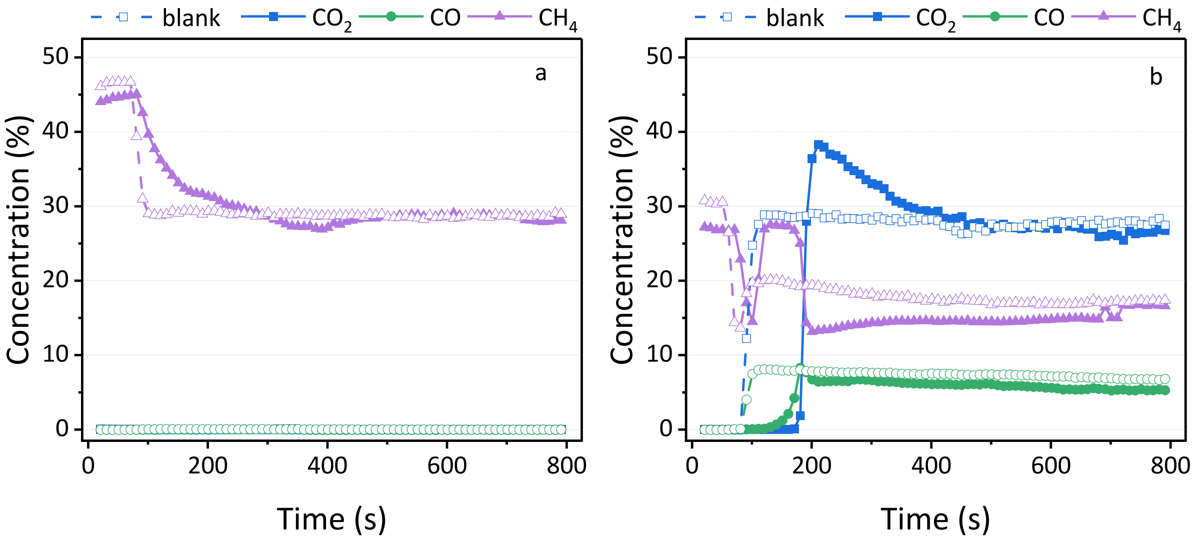

Figure SI - 9 Concentration of CO_2, CO and CH_4 in the outlet stream during a typical flow plasma in (a) CH_4 and (b) CO_2/CH_4. The solid points are for the zeolite, while the open symbols are for the blank measurements with quartz.

Figure SI - 9a presents the pure CH_4 plasma. There is a slight delay in the drop of the CH_4 concentration, due to the length of the lines. There is no CO_2 or CO measured, as expected. The CH_4 concentration drops quicker in the blank (quartz sand) measurement than in the zeolite. Perhaps some adsorption-desorption effects still play a role for CH_4, since there is no CO_2 to compete with the binding sites on the zeolite. After 500 s, they both reach the same equilibrium concentration. Since no CO_2 is present, no H_2O is formed, and the results are not included in Section 3.2.2 in the main paper.

In Figure SI - 9b, a typical DRM plasma is presented, for which the results from the humidity meter are displayed in the main paper in Section 3.2.2. To prevent any adsorption of CO_2, a quick switching of gases is needed. First, Ar/CH_4 is inserted at 40/20 mL_n/min to flush. Then, the plasma is ignited and the flow is quickly switched to $Ar/CH_4/CO_2$ 20/20/20 mL_n/min. There is a slight delay (< 30 s) in the CO_2 concentration in the blank experiment (quartz sand) due to the later addition. Interestingly, the delay is even larger in zeolite 5A, followed by a higher desorption peak around 200s. This suggests that there is still some CO_2 adsorbed on the material, followed by immediate desorption, before reaching the equilibrium concentration. At equilibrium after 600s, it seems that the CH_4 conversion is higher (i.e. lower CH_4 concentration) than the CO_2 conversion, typical for DRM reactions with a CO_2/CH_4 feed.

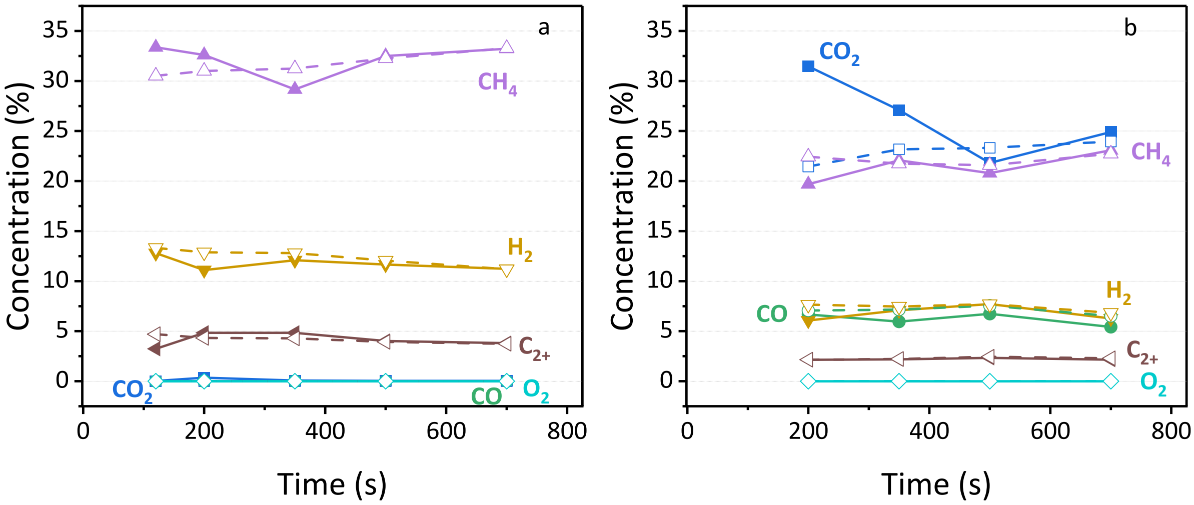

Figure SI - 10 Volume fraction of all different components identified by GC for discrete points in time in (a) a CH_4 plasma and (b) a CO_2/CH_4 plasma. The solid points are for the zeolite, while the open symbols are for the blank measurements with quartz.

The results of the GC are also in line with expectations from a typical flow plasma reactor. The CH_4 plasma shows significant H_2 and hydrocarbon production. For the CO_2/CH_4 mixture, the concentration of CO_2 is higher at first, in line with the small desorption peak that was observed in the FTIR results around 210 s, but then all concentrations reach the same equilibrium as in the blank measurement.

S10. TGA results

The thermogravimetric analysis was conducted with approximately 50 mg of material, in an air atmosphere at atmospheric pressure and a rise of 5°C/min. The results are shown in Figure SI - 11.

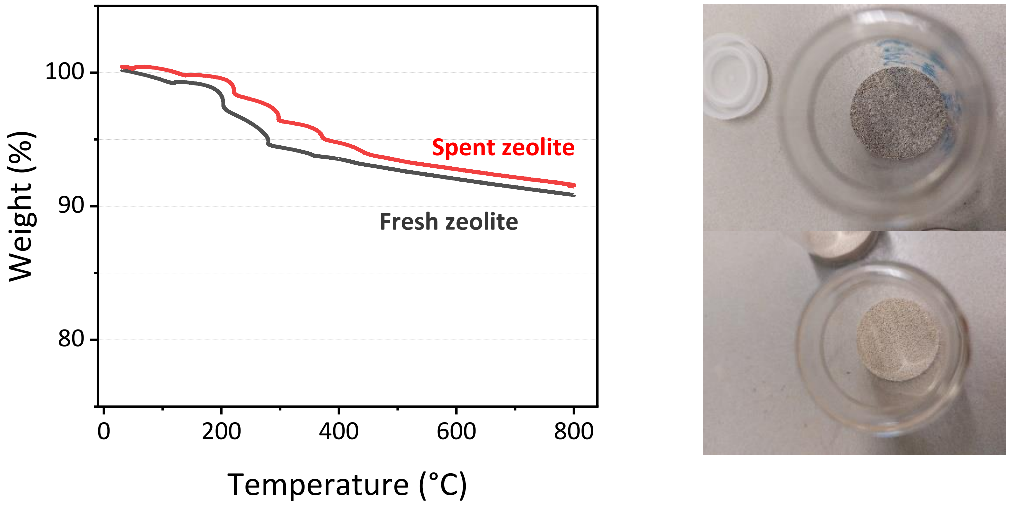

Figure SI - 11 Weight as a function of the programmed temperature in the TGA for the fresh zeolite sample and the zeolite sample after the reaction as described in the main paper. Pictures of the spent (top) and fresh (bottom) samples are included.

Visually, there is a clear difference between the fresh sample and the spent zeolite. The spent zeolite is darker in color, probably due to the carbon deposition. In the TGA, the initial profiles are very similar for the fresh and the spent material. Volatiles and CO_2 adsorbed from ambient air can play a role, as well as H_2O desorption which is known to contribute (in the range up to 400°C[5]). In the curve of the spent sample, there are two subtle peaks in the range of 300-450 °C, but the overall carbon deposition seems limited compared to the literature on TGA of zeolites with coke.[6,7]

The fresh zeolite demonstrates slightly more weight decrease than the spent sample, for which two explanations are possible. First, the fresh zeolite might contain strongly adsorbed water that is removed during the TGA procedure, while in the spent sample, the plasma previously removed the H_2O. Second, the carbon on the spent sample might block the pores so that less ambient CO_2 and H_2O could adsorb in the time between the plasma treatment and TGA. Indeed, after the plasma procedure, all CO_2 and H_2O should be desorbed from the material, and some carbon deposition is formed. However, the DBD reactor then must be opened to retrieve the material and perform the TGA in a different machine. Hence, the spent sample is open to the ambient atmosphere, but it has a slightly lower available surface for adsorption of ambient molecules compared to the fresh sample due to the carbon deposition.

References

(1) Wilson, S. M. W.; Kennedy, D. A.; Tezel, F. H. Adsorbent Screening for CO2/CO Separation for Applications in Syngas Production. *Sep Purif Technol* **2020**, *236*, 116268. https://doi.org/10.1016/j.seppur.2019.116268.

(2) Peeters, F.; Butterworth, T. Electrical Diagnostics of Dielectric Barrier Discharges. In *Atmospheric Pressure Plasma*; Anton, N., Zhiqiang, C., Eds.; IntechOpen: Rijeka, 2018; p Ch. 2. https://doi.org/10.5772/intechopen.80433.

(3) *NIST Standard Reference Database Number 69*. https://doi.org/10.18434/T4D303.

(4) Lee, D. H.; Song, Y.-H.; Kim, K.-T.; Lee, J.-O. Comparative Study of Methane Activation Process by Different Plasma Sources. *Plasma Chemistry and Plasma Processing* **2013**, *33* (4), 647–661. https://doi.org/10.1007/s11090-013-9456-6.

(5) Gómez, L.; Martínez, I.; Navarro, M. V.; García, T.; Murillo, R. Sorption-Enhanced CO and CO2 Methanation (SEM) for the Production of High Purity Methane. *Chemical Engineering Journal* **2022**, *440*, 135842. https://doi.org/10.1016/j.cej.2022.135842.

(6) Ochoa, A.; Ibarra, Á.; Bilbao, J.; Arandes, J. M.; Castaño, P. Assessment of Thermogravimetric Methods for Calculating Coke Combustion-Regeneration Kinetics of Deactivated Catalyst. *Chem Eng Sci* **2017**, *171*, 459–470. https://doi.org/10.1016/j.ces.2017.05.039.

(7) Ortega, J. M.; Gayubo, A. G.; Aguayo, A. T.; Benito, P. L.; Bilbao, J. Role of Coke Characteristics in the Regeneration of a Catalyst for the MTG Process. *Ind Eng Chem Res* **1997**, *36* (1), 60–66. https://doi.org/10.1021/ie9507336.
